# Transurethral seminal vesiculoscopy for intractable hematospermia: experience from 144 patients

**DOI:** 10.1186/s12894-021-00817-4

**Published:** 2021-03-27

**Authors:** Wei-Kang Chen, Dong-Dong Yu, Zhi-Xia Chen, Peng-Fei Li, Jian Cai, Yu-Peng Liu, Zhi-Gang Wu

**Affiliations:** 1 Department of Andrology, The First Affiliated Hospital of Wenzhou Medical University, Wenzhou, 325000 Zhejiang China; 2 School of Public Administration and Health, Wenzhou Medical University, Wenzhou, 325000 Zhejiang China; 3 Reproductive Health Research Center, Health Assessment Center of Wenzhou Medical University, Wenzhou, 325000 Zhejiang China

**Keywords:** Hematospermia, Transurethral seminal vesiculoscopy, Therapy

## Abstract

**Purpose:**

to describe the methodology of transurethral seminal vesiculoscopy and the anatomy of the area of the verumontanum, and to determine the safety of this procedure, especially in terms of postoperative complications.

**Methods:**

This retrospective observational study enrolled 144 patients with intractable hematospermia from May 2011 and August 2019. A 4.5/6.5-Fr vesiculoscope was inserted into the seminal vesicle to deal with the positive findings. The solution of quinolones was used to rinse each seminal vesicle.

**Results:**

In this study, Transurethral seminal vesiculoscopy was successfully performed in 139 patients (96.53%). Hematospermia was alleviated or disappeared in 116 (80.56%) patients by less than half a year after surgery. Common intraoperative manifestations were hemorrhage, stones, utricle polyps and cysts. The surgical approach in our study were categorized into four types, including 24 (16.7%), 73 (50.7%), 42 (29.2%), and 5 (3.5%) cases in Type A (natural opening of the ejaculatory duct), B (trans-duct fenestration), C (trans-utricle fenestration), and D (not founded), respectively. Sexual function change was recorded in 12 patients of 111 patients, all by the method of trans-utricle fenestration, including 8 (7.21%), 3 (2.70%), and 1 (0.90%) patients in shorter intravaginal ejaculatory latency time, worse erection hardness and loss of orgasm, respectively.

**Conclusion:**

Transurethral seminal vesiculoscopy is an effective and safe procedure for the management of hematospermia. The anatomy of the distal seminal tract should be understood more deeply and Wu’method (uncover-curtain method) needs to be promoted to verify its universality and safety. Besides, the complications of the function dysfunction should be discussed in the future in multi-center clinical trials.

**Supplementary Information:**

The online version contains supplementary material available at 10.1186/s12894-021-00817-4.

## Introduction

Hematospermia is the presence of blood in the semen. There are various etiologies, including inflammation, duct obstruction, cysts, tumors, vascular abnormalities, systemic factors and iatrogenic causes. In the present study, most hematospermia was self-limiting and could resolve with appropriate antimicrobial treatment [1]. Nevertheless, there remain some patients who suffered recurrent hematospermia, for which the conservative management is not effective. This is a condition that causes great anxiety and panic among patients, leading to damaged physical and mental health [2].

Current non-invasive imaging examinations used for the diagnosis of hematospermia include transrectal ultrasonography (TRUS), pelvic computed tomography (CT) and magnetic resonance imaging (MRI). Whereas examinations can diagnose, they may miss some small lesions or produce false positives. Transurethral seminal vesiculoscopy (TUSV) can achieve both diagnosis and therapy and more and more operators have successfully performed this operation with good effect [3–5]. Nevertheless, the long-term efficacy and safety have not been reported, especially regarding postoperative complication of sexual dysfunction. Furthermore, the procedure of the TUSV has not been standardized, because of unfamiliarity with the anatomy of the area of the verumontanum.

Here, we summarize our experience from 144 TUSV patients by illustrating detailed surgical methods, treatment outcomes, and intraoperative findings, with a discussion on the postoperative sexual function change and kinds of operative approaches. We supposed that transurethral seminal vesiculoscopy is an effective and safe procedure for the management of hematospermia, but still needs our future researches.

## Materials and methods

### Study cohort

This study enrolled consecutive patients who had intractable hematospermia and underwent surgery of TUSV between May 2011 and August 2019 in the Department of Urology, The First Affiliated Hospital of Wenzhou Medical University, Wenzhou, Zhejiang Province, China. All patients presented with continuous or episodic hematospermia.

Regarding the diagnosis of hematospermia, we mainly rely on detailed consultation and photos of semen provided after patients’ sex life for reference. Then, they would take a series of physical examination, laboratory test (serum PSA, coagulation profile, blood routine, urine routine, liver function test, kidney function test) and imaging examination (TRUS or MRI) to exclude other diseases and confirm the diagnosis (An additional picture file shows this in more detail [see Additional file 1]). In addition, some patients would provide corresponding semen examination reports. These patients who still have obvious symptoms of hematospermia with the naked eye, after taking antibiotics (usually quinolones) regularly for at least 4 weeks, physical therapy and lifestyle improvements for more than 3 months, were diagnosed as continuous or intractable hematospermia. The specific diagnosis and treatment plan was presented in Additional file 2. Finally, patients who asking for further treatment, and then they were admitted to be treated by TUSV after comprehensive doctor–patient communication, who are enrolled in this study.

Approval for this study was granted by the Ethics Committee of The First Affiliated Hospital of Wenzhou Medical University, Wenzhou, Zhejiang Province, China and written informed consent was obtained from all patients before administering treatment. All methods were carried out in accordance with relevant guidelines and regulations.

### Surgical procedures

**Fig. 1:**
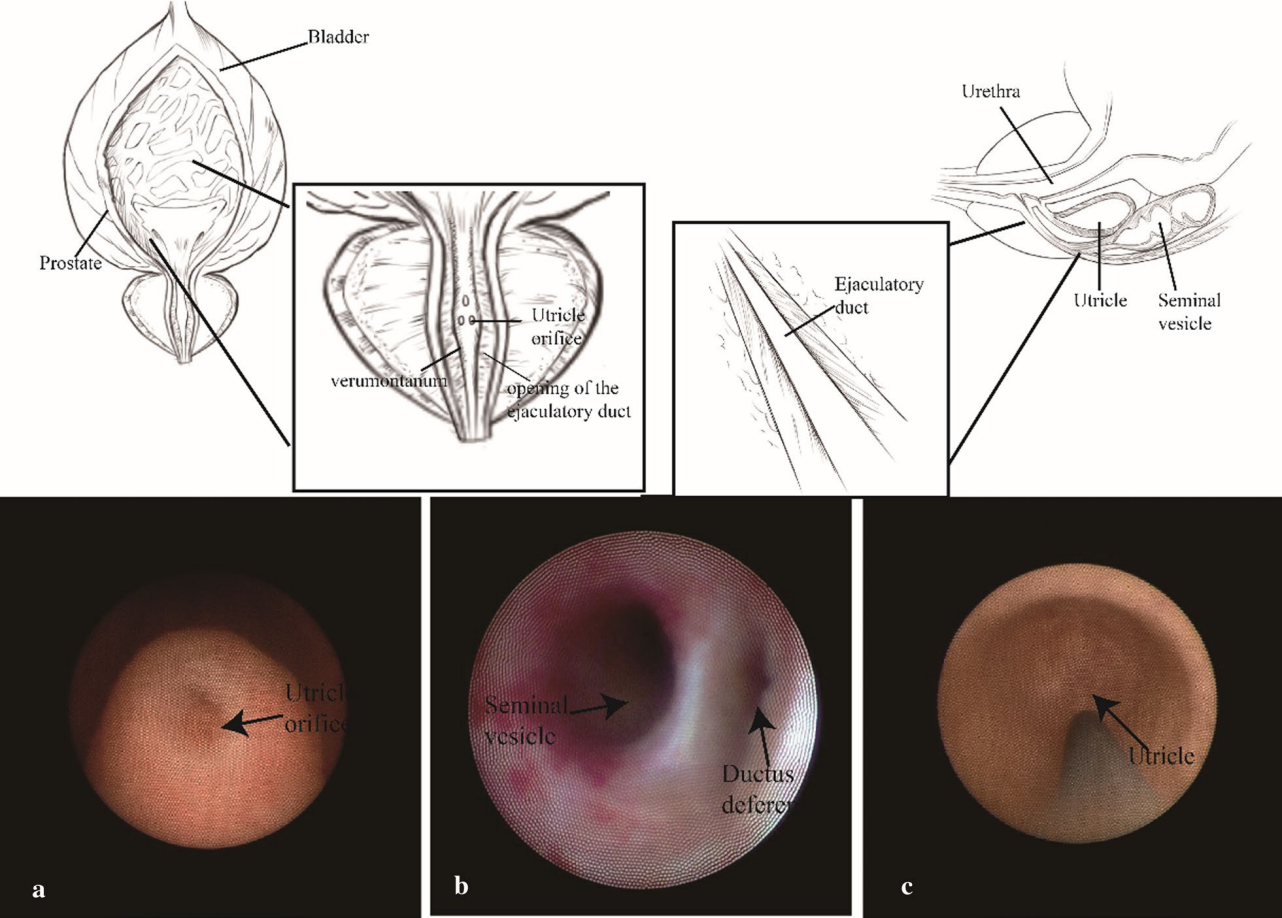
Anatomy of the verumontanum, utricle and ejaculatory duct

Each patient was placed in the lithotomy position and received general anesthesia. A small-diameter rigid vesiculoscope (4.5/6.5-Fr, Wolf, Germany) was inserted into the normal anatomic path of the urethra. After passing through two bends of urethra, the verumontanum was shown in the prostatic urethra. The anatomy of the distal seminal tract and its surroundings are noted in Fig. 1 and the subsequent procedure is illustrated in Additional file 3.

**Fig. 2:**
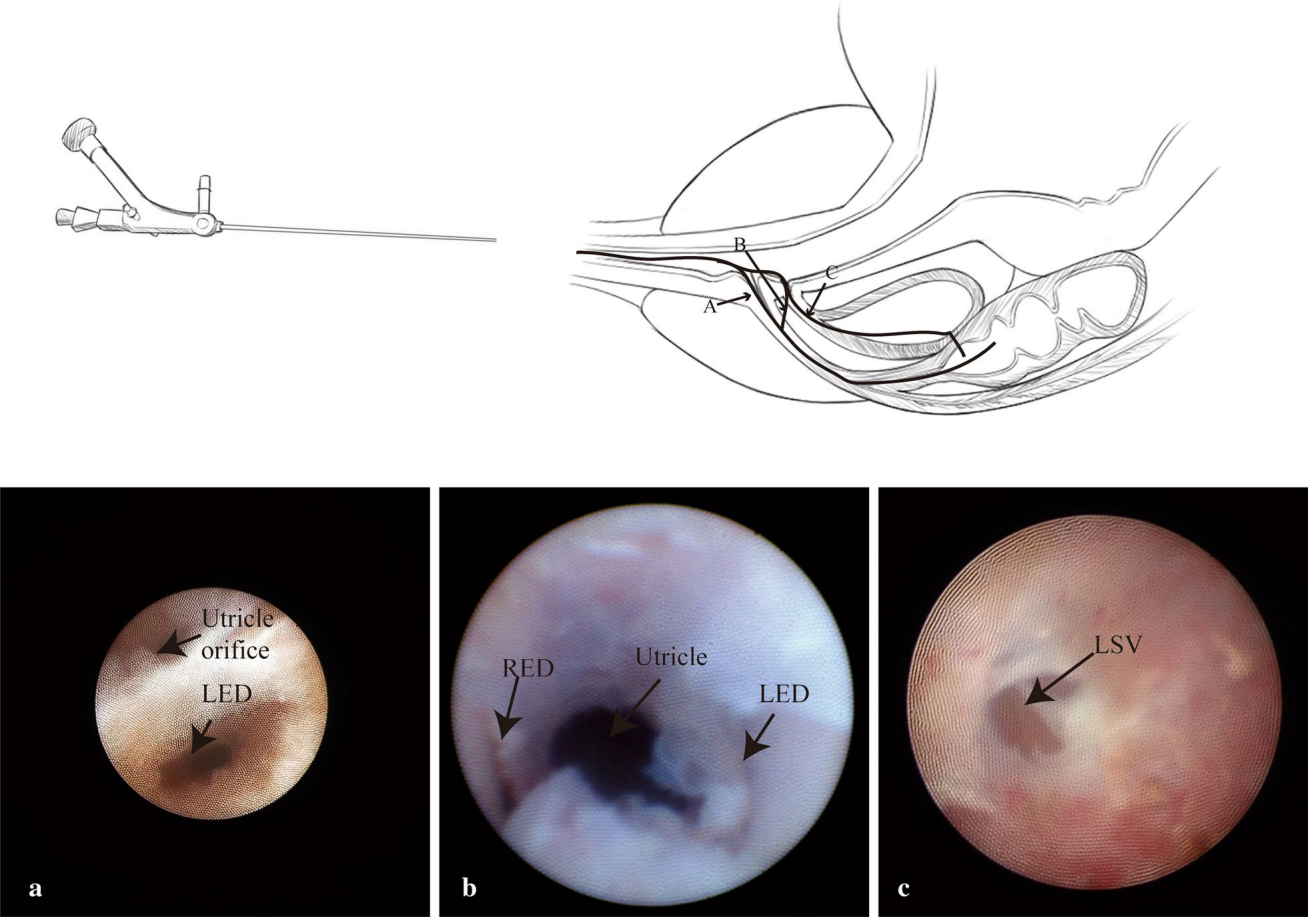
Approach through seminal vesicle. Arrow A, approach through the opening of ejaculatory duct. Arrow B, approach through the bilateral inner wall of the entrance of utricle. Arrow C, approach through the lateroposterior aspect of the utricle wall. LED, left ejaculatory duct. RED, right ejaculatory duct. LSV, left seminal vesicle

There are three ways to enter the seminal vesicle in our study (Fig. 2). In some patients, the opening of the ejaculatory duct was easily found at a position lateral to the verumontanum using a hand-controlled irrigation pressure change, but it usually was found covered with white membranous tissues. The type A way is to through the founded natural opening of the ejaculatory duct. When the path to the ejaculatory duct was not be distinguished on the preface of verumontanum (Type B, C, D), a surgical approach was established. First, a small-diameter rigid vesiculoscope was inserted into the utricle orifice and then the full view of the utricle was shown on the endoscopic monitor. Second, the vesiculoscope was turned back to the opening of utricle, and tentative scratch was made on the bilateral inner wall of the entrance of utricle, approximately in the 5 o’clock and 7 o’clock position. The ejaculatory duct is just beneath the thin layer tissues and the vesiculoscope could be inserted into the ejaculatory duct gently (trans-duct fenestration, Type B); in other patients, the hidden ejaculatory duct could not be found. In Type C patients, the vesiculoscope should be inserted into the utricle. Then, the monitor should focus on the lateroposterior aspect of the utricle wall trying to find the membranous junction between the prostate utricle and the seminal vesicle. In few patients, there was a blue translucent membrane, while in most patients the junction could not be found easily. The trick for identifying the junction is to rhythmically control saline irrigation and watch carefully. The membranous junction often appeared in a periodic motion of sinking and bulging under the change of water pressure. Finally, a fenestration could be made by puncturing using a guidewire (Straight Tip, stiff shaft guidewire, NGCV0557, BARD, NICORE, U.S.A.) through seminal vesicle (trans-utricle fenestration). The typical appearance of the seminal vesicle (SV) is similar to honeycomb. However, in some patients, these pathways could not be found (Type D), and the operation through the seminal vesicle failed.

Finally, regardless of pathway to the SV, the surgeon needed to go through every small cavity in the SV. Then, kinds of treatments were performed according to the endoscopic findings of the seminal vesicle. For patients who had large SV stones that could not easily be washed out using continuous saline irrigation but keeping the pressure of SV as low as possible, holmium laser lithotripsy was performed. After this procedure, small crushed stones could be flushed out. When old or new hemorrhage was found in the SV, the subsequent step was to wash it out. Until no stone and hemorrhage could be found in SV, 250 ml solution of quinolones (typically 250 mL of saline solution with 400 mg of Mosifloxacin) would be used to rinse each SV. Meanwhile, the same method was performed for cleaning up utricle. Finally, in patients who underwent trans-utricle fenestration, the surgical path between the prostatic utricle and seminal vesicle was expanded using the scope body or a holmium laser incision to prevent possible stenosis and recurrence, while patients who underwent trans-duct fenestration accepted expanding for new paths by the scope body. After the operation, a 14-Fr Foley catheter was retained for 24 h. Besides, within 48 h after surgery, patients still were infused with intravenous antibiotics (always 250 mL of saline solution with 400 mg of Mosifloxacin) once a day. Before leaving hospital, some patients had MRI to ensure that the operation was successful (large stones or blood clots disappeared compared with pre-operative MRI).

### Data collection and statistical methods

We retrospectively recorded demographic and operation data, including age, disease duration, and basic disease history. Therapeutic outcomes were recorded. Patients whose semen turned back to milky white or presented no red blood cells under the microscope were regarded as cured or disappeared; Patients whose semen changed from dark brown or bright red to light red or milky white but presented some red blood cells under the microscope were regarded as reduced. We followed up three times at 3 months, 6 months and 12 months after surgery. We asked the patient's situation of hematospermia (disappeared or present) and sexual function (i.e. intravaginal ejaculatory latency time and erection hardness scale) at every follow-up interview by call. Continuous variables such as age were presented as mean (Std.), except follow-up period expressed as median (range). Categorical variables such as hypertension were expressed as counts and percentages. The comparison of efficient of TUSV in two kind of patients were used by Chi-square test. All analyses were conducted using Stata 13.0 (Stata Corp, College Station, TX, USA).

## Results

### Preoperative data and treatment outcomes

A total of 199 patients presented to the urology and andrology department for recurrent hematospermia between 2011 and 2019; however, only 144 patients were enrolled in this study. The specific include/exclude process could be seen in (Additional file 4. The characteristics are shown in Table 1. The effect of TUSV was not immediate. Hematospermia disappeared or reduced in 98 (68.06%) and 18 (12.50%), respectively, by no more than 3 months after surgery (most symptoms disappear within a month). Six of 98 patients experienced recurrence by 12 months after surgery. Symptoms persisted and aggravated in 22 (15.28%) and 6 (4.17%) patients, respectively. The treatments for patients who were dissatisfied with the outcome were always antibiotics and lifestyle improvement; nevertheless the effectiveness remained inadequate. The results of MRI all showed that blood clots or stones disappeared in the seminal vesicles and utricle.

**Table 1:** Characteristics of patients

	Patient with hemospermia (n = 144)	
Age (years)	42 (30.73–52.25)	
Duration of hemospermia (months)	12 (12–24)	
Hypertension	24/144 (16.67%)	
DM	2/144 (1.39%)	
Chronic hepatitis B	7/144 (4.86%)	
*Urine routine*		
Microscopic hematuria	39/141 (27.46%)	
Pyuria	3/141 (2.11%)	
Proteinuria	6/141 (4.23%)	
Sugaruria	1/141 (0.70%)	
Not tested	n = 3	
PSA(ng/ml)	0.72 (0.49–1.13)	
*TRUS/MRI fingding*		
Dilatation or enlargement of SV	70/141 (49.65%)	
Hemorrhage	20/141 (14.18%)	
Inflammation of SV	25/141 (17.73%)	
Overall cyst	52/141 (36.88%)	
Prostate	22/141 (15.60%)	
Mullerian duct	26/141 (18.44%)	
Seminal vesicle	3/141 (2.13%)	
Ejaculatory duct	4/141 (2.84%)	
Prostate	74/141 (52.48%)	
Hyperplasia	42/141 (26.95%)	
Enlargement	25/141 (15.60%)	
Inflammation	7/141 (3.55%)	
Stone	5/141 (1.42%)	
Nodular	2/141 (0.71%)	
Normal	10/141 (7.09%)	
Not tested	n = 3	
Successful TUSV	139/144 (96.53%)	
Follow-up period (days)	1021.5 (72–2626)	

Continuous variables expressed as median (interquartile range), except follow-up period expressed as median (range)

### Surgical approach and intraoperative findings

**Fig. 3:**
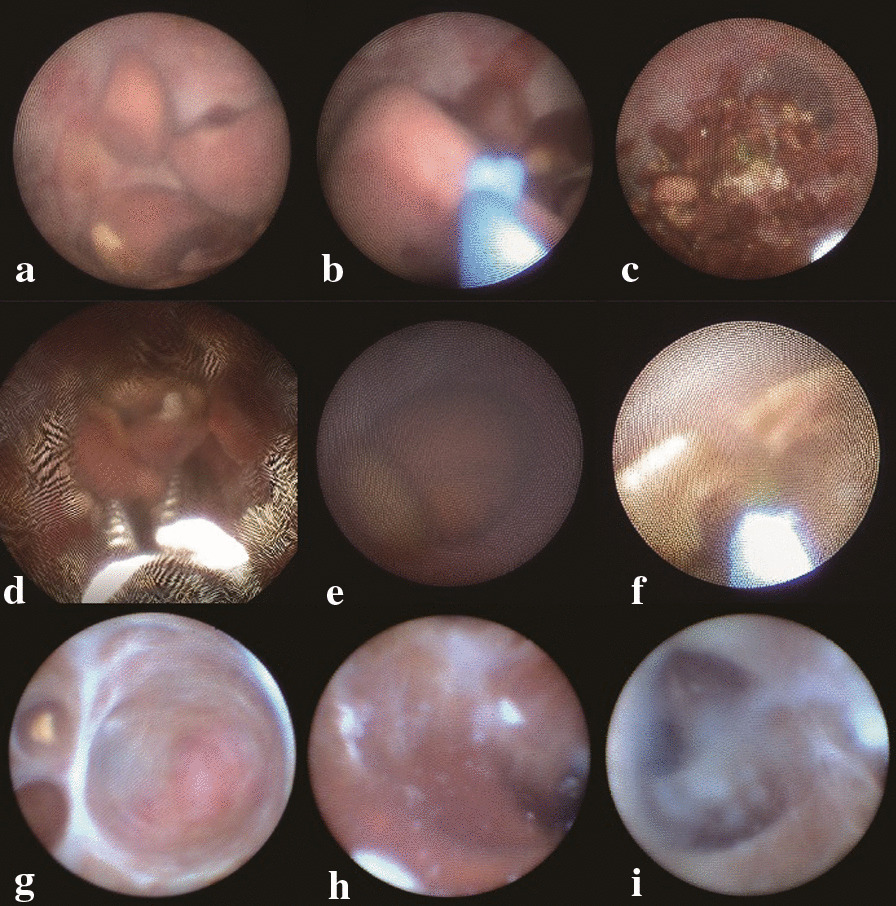
Perioperative findings in transurethral seminal vesiculoscopy. **a** Stones in the utricle; **b** thulium fiber laser applied to crush stones in the utricle; **c** crushed stones; **d** grasping forceps applied for removal of stones in the utricle; **e** big stones in the seminal vesicle; **f** thulium fiber laser applied to crush stones in the seminal vesicle; **g** small stone in the seminal vesicle; **h** old hemorrhage in the seminal vesicle; **i** seminal plasma in the seminal vesicle

The surgical approaches in our study were categorized into four types, including 24 (16.67%), 73 (50.69%), 42 (29.17%) and 5 (3.47%) patients in Type A, B, C, and D, respectively. Normally, there are nothing in SV, utricle or ED; however, in patients who underwent this surgery, hemorrhage, stones and cyst in the vision (SV, utricle, or both areas) were presented in 75 (52.08%), 72 (50.00%) and 53 (36.81%) patients, respectively. Utricle polyp was observed in only one patient. Nothing positive occurred in 24 (16.67%) patients (Fig. 3).

The rate of hematospermia disappeared and reduced in patients with different intraoperative findings.

The rate of hematospermia disappeared and reduced in patients whose intraoperative findings were only hemorrhage was 88.00% (22/25), while in patients whose intraoperative findings were both hemorrhage and stones was 73.17% (30/41) (*p* > 0.05).

### Complications

Complications were as follows: Subjective semen volume decreased in two patients without semen analysis, both of whom underwent TUSV by inserting into the ejaculatory duct opening and refused to have a further medical examination. Every patient was asked about the sexual function before and after operation; however, only 111 patients accepted and answered this question. Of 111 patients, sexual function change was presented in 12 (10.81%), including 8 (7.21%), 3 (2.70%) and 1 (0.90%) patients who reported shorter sexual time, worse erection hardness and loss of orgasm, respectively (Table 2). Of eight patients, five complained of shorter postoperatively self-estimated intravaginal ejaculatory latency time (IELT); however, all their IELT before and after operation was longer than five minutes. However, two patients indicated their IELT changed from longer than 5 min to shorter than 2 min after surgery, which may accord with the clinical standard of premature ejaculation (PE). One patient represented that his IELT was worse, while his IELT had been shorter than 2 min before surgery. Of three patients, all reported their erection hardness scale (EHS) changed from IV to III. All 12 patients denied a history of taking testosterone modulating medications. The remaining 99 patients reported no change in their sexual function. As for other complications, only one patient with postoperative epididymitis was treated with a week of intravenous quinolone antibiotics. No retrograde ejaculation, urinary reflux into the ejaculatory duct, fistula, or other severe complications were observed.

**Table 2:** Sexual function change of patients before and after TUSV

	Preoperation	Postoperation	Patients (n = 144)
Intravaginal ejaculatory latency time (minutes)	> 5	> 5	5
	> 5	< 2	2
	< 2	< 2	1
Erection hardness scale	IV	III	3
Frequency of orgasm	HIGH	Low	1

## Discussion

Since the first application of endoscopy for examining the interior of the seminal vesicle ex vivo by Shimada et al. [6] in 1996, this new method for observing SV directly had attracted the attention of the andrologists and urologists. Nowadays, the vesiculoscope has been used in some large hospitals. However, there remain many surgeons beginning to operate this procedure, for whom transurethral seminal vesiculoscopy (TUSV) is an advanced but challenging procedure. The present study reported some tricks and details devoting to how to use TUSV, but it is not sufficient enough. Therefore, we focused on sharing our experience and putting forward some views after at least 10 years’ application of TUSV.

Transurethral seminal vesiculoscopy has been recommended as the first-line diagnostic modality [7] in hematospermia patients because of its real-time images and low cost. Besides, TUSV is also helpful for therapy.

Xing et al. [8] concluded that TUSV was superior to TRUS for diagnosis, especially for lesions with stones and obstruction. It can also be used to treat recurrent hematospermia and ejaculatory duct obstruction [9] caused by stones or cysts by facilitating removal of calculi, elimination of obstruction and drainage of infection. Liu et al. [3] reported that the hematospermia symptom in 94.4% patients was completely treated and alleviated. Liu et al. [10] reported that hematospermia was alleviated in 89% patients. During the long history of application, it was confirmed to be an effective procedure for both diagnosis and treatment, without severe complications.

In the current study, the improvement rate of hematospermia was 80.56% and the recurrence rate was 6.12%, comparable to similar studies reported in the literature [4, 5]. The reason why our improvement rate of hematospermia was relatively lower than other studies may be the relatively longer duration of hematospermia in enrolled patients. In the meantime, to our knowledge, our median follow-up period of 3.38 years was the longest. For this reason, we are surprised that there were no long-term complications. Our findings provide more powerful evidence supporting the safety and efficiency of TUSV.

We would like to highlight two additional points:

First, we want to name one of approaches of TUSV (*Wu’ method*) to capture the attention of surgeons and to promote application of this method (An additional movie file shows this in more detail [see Additional file 5]). In the present articles all over the world, we find four ways to treat hematospermia surgically, including a method of transurethral resection of the ejaculatory duct (TURED) and three methods of TUSV. In our concept, we don’t divide TURED into TUSV, due to differences of surgical instrument of vesiculoscope (usually 24-Fr vs. 4.5/6.5-Fr; combine with an electrocautery loop vs. only a scope) and surgical procedure (resection vs. only blunt operation) [11].

We are trying to describe four methods of treating hematospermia as follows: when the opening of the ejaculatory duct is observed from the urethra, inserting into this natural orifice (the first way) is the first choice; whereas in other patients, when the opening is not identified clearly, there are two methods.

First, some surgeons applied TURED (the second way) to observe the covered ejaculatory duct openings. The probable complications of retrograde ejaculation and urinary reflux limited the widely application of this method. At present, surgeons hardly use TURED to locate the opening of the ejaculatory duct exclusively, while some surgeons try to treat patients with Mullerian duct cyst [12]. In addition, surgeons can insert into the verumontanum orifice and establish a surgical path through the ejaculatory duct (the third way) or SV (the fourth way) directly. The only difference between these two approaches is the puncture site (the inner wall of the utricle entrance or the laterposterior aspect of the utricle wall). Anyway, the most important factors are safety and efficacy depending on which method should be used firstly.

We can evaluate safety on the basis of anatomical structure. Nguyen et al. [13] reported the ejaculatory duct and SV consist of three similar histological layers, including a columnar epithelium, a collagenous coat and a muscular layer. The most surprising finding was that all these three layers become thin as the duct proceeds distally, especially the almost disappearing muscular layer. It is not difficult to conclude that the more distal area we puncture, the fewer tissues we destroy. Li et al. [14] reported that the opening of the ejaculatory duct was covered by the valves that needs to be protected in order to prevent urinary reflux into the ejaculatory ducts. This finding may explain why TURED causes these complications of retrograde ejaculation and urinary reflux.

After approximately 10 years’ application of rigid vesiculoscope, we recommend an approach (*Wu’method*) through the distal ejaculatory duct, not inserting into the opening of the ejaculatory duct, passing the inner wall of the utricle entrance. The key procedure of this method is to tentatively scratch the entrance of the inner wall of the utricle entrance using the former endoscope. Then, the membrane-like tissue is identified under the monitor. After setting up this tissue, the passageway can be detected and used for entering the ejaculatory duct. Because of the process of the creating and setting up the membrane-like tissue, similar to the action of setting up a curtain or quilt, we would like to call this method the *uncover-curtain method*. In our experience, we found that the success rate of this method was high and the outcomes were good. We emphasize this approach by detailed introduction of seminal tracts and try to make a standard for TUSV so as to encourage its use.

Second, the complications of sexual function are important to discuss. To the best of our knowledge, this study is the first research paying attention to sexual dysfunction after this surgery. The follow-up results showed that 12 patients seemed to suffer sexual dysfunction, including the tendency of premature ejaculation and erectile dysfunction. After we investigated the specific surgical approaches of these 12 patients, we surprisingly discovered that all of them had been treated by puncturing using a guidewire through the SV. This fact triggers our thinking to one guess whether this approach would cause more sexual function influence than other two approaches. Sexuality, as an inherent need for human beings [15], attracts the attention of both patients and doctors. For this reason, we should concentrate on researching the potential relationship between the operation by puncturing using a guidewire through SV and sexual dysfunction. In case this kind of operation can cause sexual dysfunction, we should identify the specific mechanism and countermeasures to prevent these complications by abandoning or improving this method of TUSV. Therefore, our next purpose is to prepare a study to investigate this potential relationship.

There are some limitations in this study. First, its retrospective design invites recall bias. The results of the postoperative outcome are judged by the view of the patients solely. Besides, most patients lacked semen analyses both before and after surgery, due to most patients (always have one or more kids) without the desire to fertile and the expensive price of semen related tests. However, we will collect some younger patients and acquire their semen analyses before and after surgery in the future, because of its effect to verify validity of TUSV. Second, the situation of sexual dysfunction lacks of evaluation of some scales and other examinations, including IIEF-5 or penile Doppler USG investigation. Third, hematospermia is usually a self-limiting disease and the natural course might interfere with the complete remission and improvement rates. However, in this study, patient who have a long history of hematospermia and have been ineffective after long-term treatment were enrolled. In addition, of 16 patients who refused surgery (10 patients before 2011 and 6 patients in this study), only 1 patient recovered within 3 years after a long-term follow-up. Although the probability of natural recovery of our patients is low, it cannot be ruled out that a small number of patients were cured by themselves. Fourthly, we found that the performing of TUSV in patients with single intraoperative finding (only hemorrhage) was more effective than performing in patients with various intraoperative findings (both hemorrhage and stones), which perfectly meet clinical prognosis. However, *p* > 0.05, because of the few number of total cases. Therefore, further verification is needed.

## Conclusions

TUSV may be an effective and safe procedure for treating recurrent hematospermia by blocking the vicious cycle of stasis, stones and seminal vesiculitis. The anatomy of the distal seminal tract should be understood more deeply and *Wu’method* (*uncover-curtain method*) needs to be promoted to verify its universality and safety. Besides, the complications of the function dysfunction should be discussed in the future in multi-center clinical trials.

## Supplementary Information

**Additional file 1**. Abnormal appearances in patients’ MRI. The blue arrow points to the seminal vesicle cyst; the red arrow points to the stone in the right seminal vesicle. MRI, magnetic resonance imaging.

**Additional file 2**. The specific plan of diagnosis and treatment. BP, blood pressure. PSA, prostate-specific antigen. TRUS, transrectal ultrasonography. MRI, magnetic resonance imaging.

**Additional file 3**. Flowchart of the surgery. For each key step in the surgery, “positive or negative” findings together with further guidance of the next move are listed in the flowchart.

**Additional file 4**. Flow-chart of the participants’ selection.

**Additional file 5**. The movie of Wu’s method.The surgeon tentatively scratches the entrance of the inner wall of the utricle entrance using the former endoscope. Then, the membrane-like tissue is identified under the monitor. After setting up this tissue, the passageway can be detected and used for entering the ejaculatory duct.

## Data Availability

All of the data are from the First Affiliated Hospital of Wenzhou Medical University. The datasets generated and analysed during the current study are not publicly available due patient privacy issues (The raw data includes the patient's name and phone number) but are available from the corresponding author on reasonable request (Zhi-Gang Wu mobile phone: + 86 13676467276; E-mail: andrologywzg@wmu.edu.cn).

## Abbreviations

TRUS: Transrectal ultrasonography
CT: Pelvic computed tomography
MRI: Magnetic resonance imaging
TUSV: Transurethral seminal vesiculoscopy
SV: Seminal vesicle
PE: Premature ejaculation
IELT: Intravaginal ejaculatory latency time
EHS: Erection hardness scale
