# Supplementary material for: Transurethral seminal vesiculoscopy for intractable hematospermia: experience from 144 patients
Source: BMC Urol. 2021 Mar 27;21:48. doi: 10.1186/s12894-021-00817-4 (PMC8005245; doi:10.1186/s12894-021-00817-4)

Additional file 1.tiff

Title of data: Abnormal appearances in patients’ MRI.

Description of data: The blue arrow points to the seminal vesicle cyst; the red arrow points to the stone in the right seminal vesicle. MRI, magnetic resonance imaging.
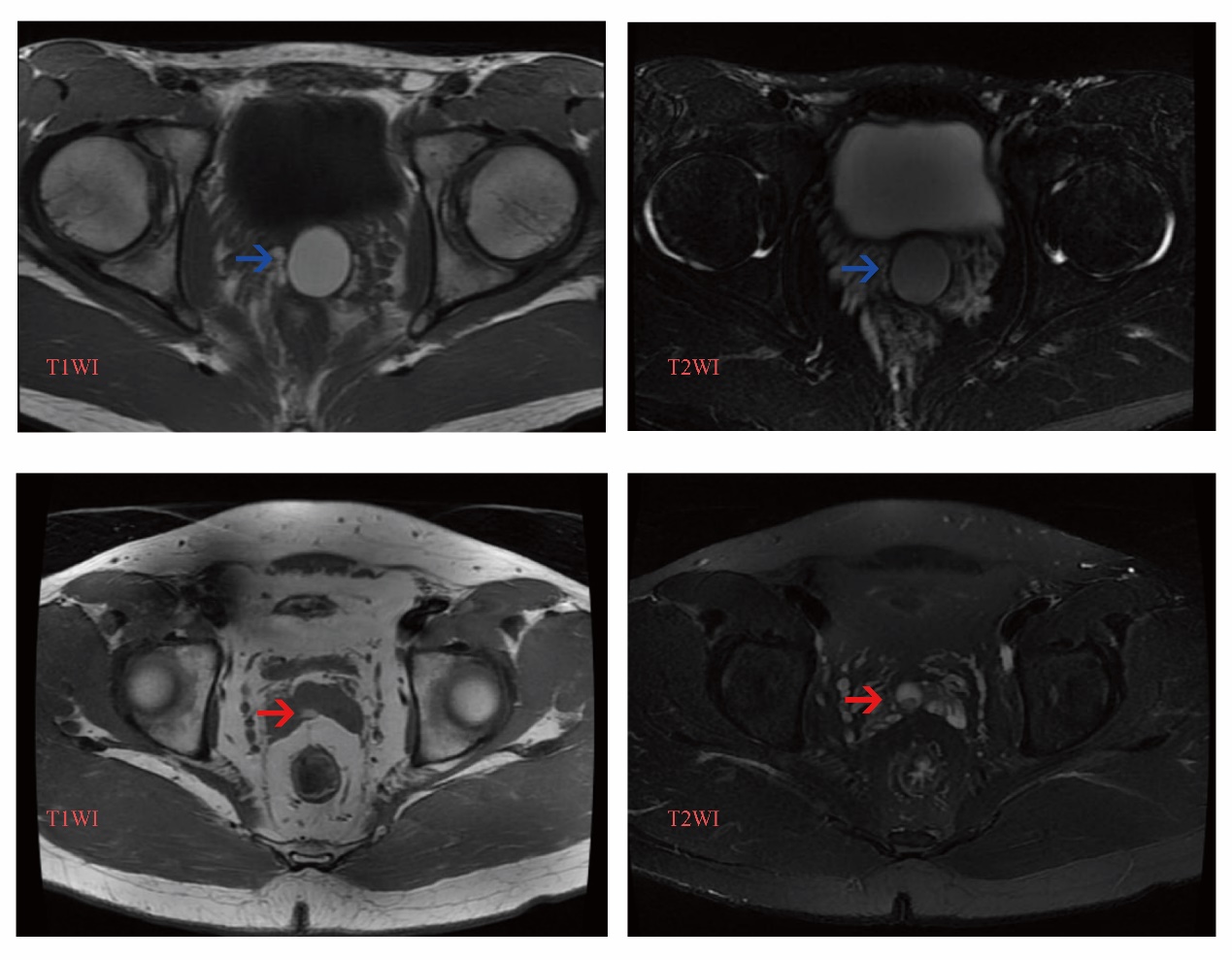

Supplement: Supplementary file 1 — Additional file 1. Abnormal appearances in patients’ MRI. The blue arrow points to the seminal vesicle cyst; the red arrow points to the stone in the right seminal vesicle. MRI, magnetic resonance imaging. [file 12894_2021_817_MOESM1_ESM.docx]
