# Supplementary material for: Transurethral seminal vesiculoscopy for intractable hematospermia: experience from 144 patients
Source: BMC Urol. 2021 Mar 27;21:48. doi: 10.1186/s12894-021-00817-4 (PMC8005245; doi:10.1186/s12894-021-00817-4)

Additional file 2.tiff

Title of data: The specific plan of diagnosis and treatment.

Description of data: BP, blood pressure. PSA, prostate-specific antigen. TRUS, transrectal ultrasonography. MRI, magnetic resonance imaging.


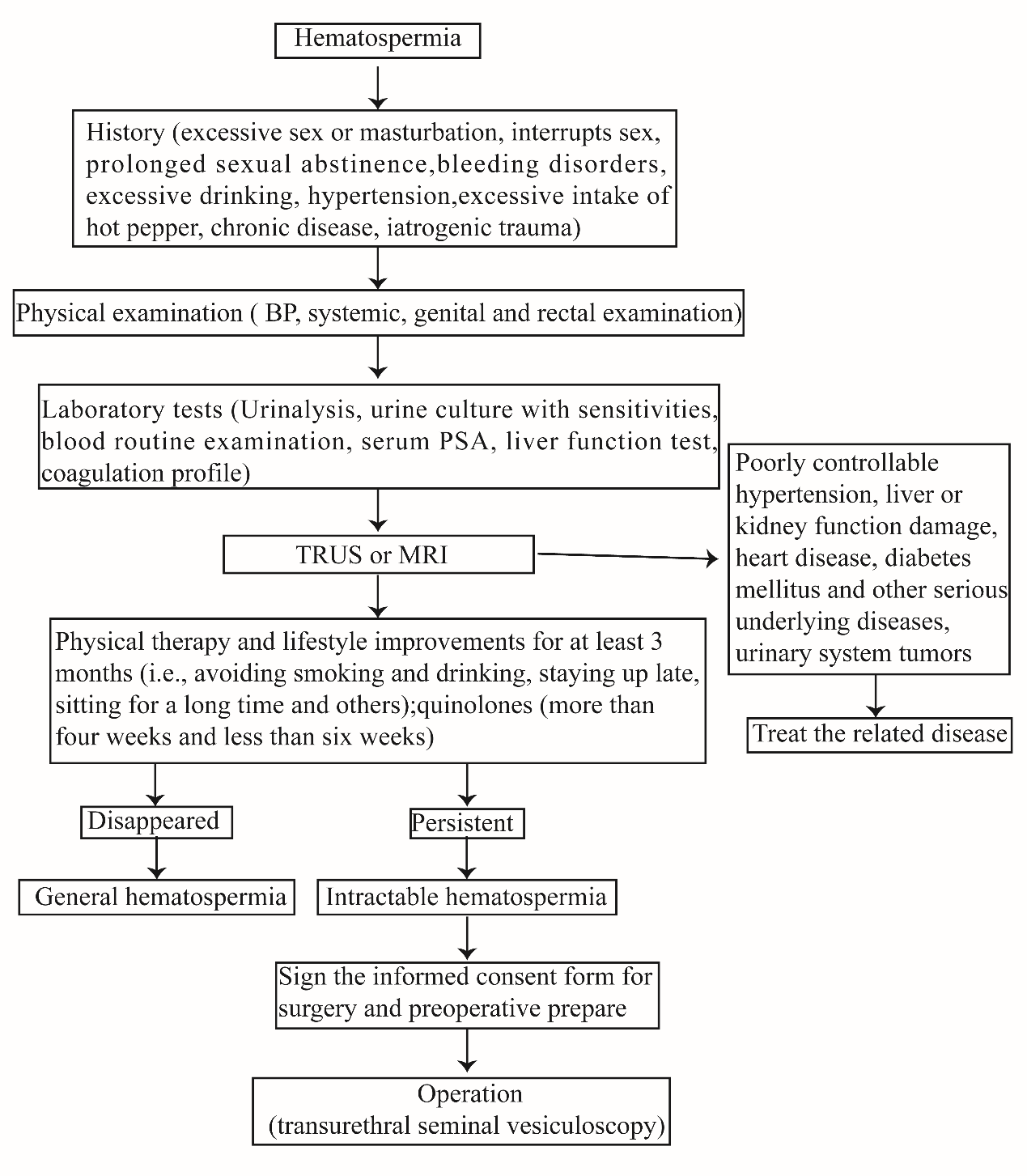

Supplement: Supplementary file 2 — Additional file 2. The specific plan of diagnosis and treatment. BP, blood pressure. PSA, prostate-specific antigen. TRUS, transrectal ultrasonography. MRI, magnetic resonance imaging. [file 12894_2021_817_MOESM2_ESM.docx]
