# Supplementary material for: Transurethral seminal vesiculoscopy for intractable hematospermia: experience from 144 patients
Source: BMC Urol. 2021 Mar 27;21:48. doi: 10.1186/s12894-021-00817-4 (PMC8005245; doi:10.1186/s12894-021-00817-4)

Additional file 3.tiff

Title of data: Flowchart of the surgery.

Description of data: For each key step in the surgery, “positive or negative” findings together with further guidance of the next move are listed in the flowchart.


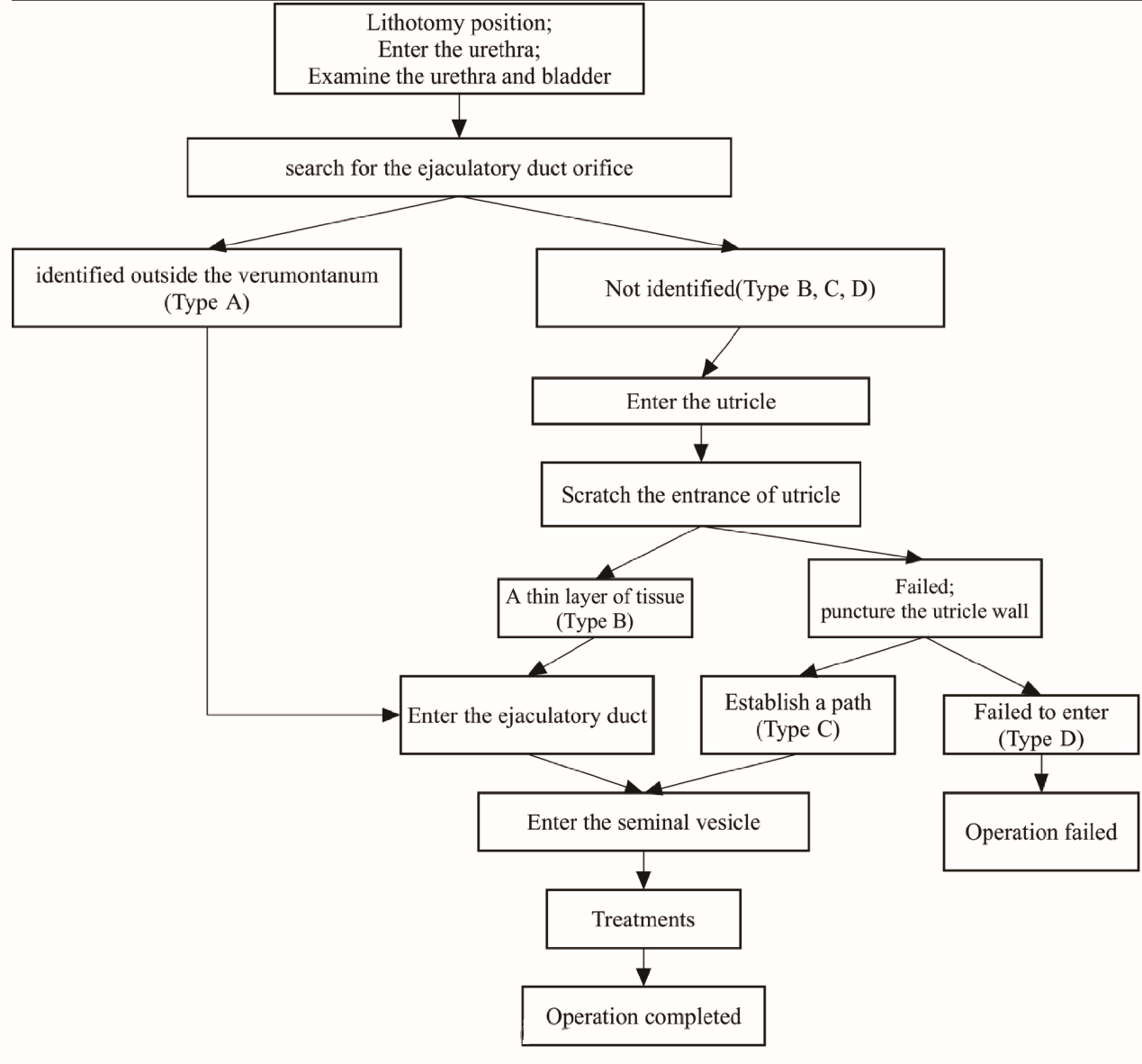

Supplement: Supplementary file 3 — Additional file 3. Flowchart of the surgery. For each key step in the surgery, “positive or negative” findings together with further guidance of the next move are listed in the flowchart. [file 12894_2021_817_MOESM3_ESM.docx]
