# Supplementary material for: Transurethral seminal vesiculoscopy for intractable hematospermia: experience from 144 patients
Source: BMC Urol. 2021 Mar 27;21:48. doi: 10.1186/s12894-021-00817-4 (PMC8005245; doi:10.1186/s12894-021-00817-4)

Additional file 4.tiff

Title of data: Flow-chart of the participants’ selection.

Description of data: None.


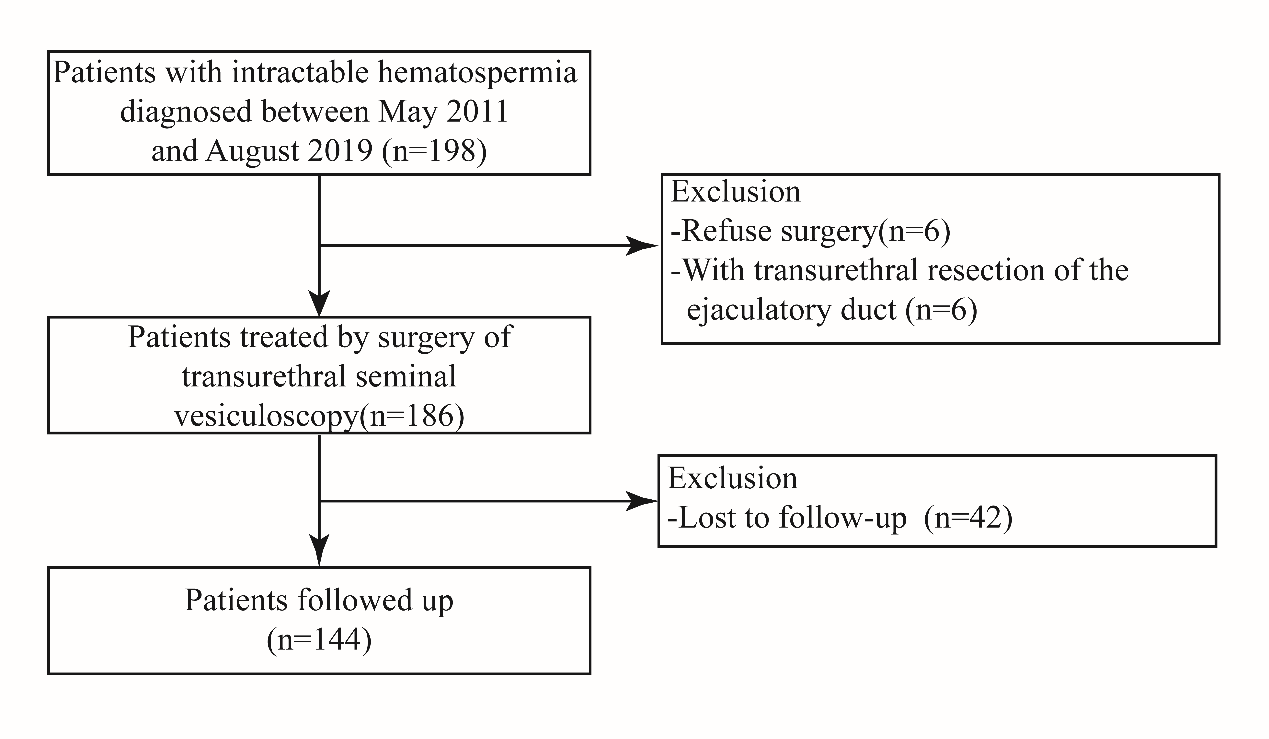

Supplement: Supplementary file 4 — Additional file 4. Flow-chart of the participants’ selection. [file 12894_2021_817_MOESM4_ESM.docx]
