# Supplementary material for: Transurethral seminal vesiculoscopy for intractable hematospermia: experience from 144 patients
Source: BMC Urol. 2021 Mar 27;21:48. doi: 10.1186/s12894-021-00817-4 (PMC8005245; doi:10.1186/s12894-021-00817-4)
Supplement: Supplementary file 5 — Additional file 5. The movie of Wu’s method.The surgeon tentatively scratches the entrance of the inner wall of the utricle entrance using the former endoscope. Then, the membrane-like tissue is identified under the monitor. After setting up this tissue, the passageway can be detected and used for entering the ejaculatory duct. [file 12894_2021_817_MOESM5_ESM.docx]

Additional file 5.mp4

Title of data: The movie of Wu’s method.

Description of data: The surgeon tentatively scratches the entrance of the inner wall of the utricle entrance using the former endoscope. Then, the membrane-like tissue is identified under the monitor. After setting up this tissue, the passageway can be detected and used for entering the ejaculatory duct
